# Early Detection and Surveillance of the SARS-CoV-2 Variant BA.2.86 — Worldwide, July–October 2023

**DOI:** 10.15585/mmwr.mm7243a2

**Published:** 2023-10-27

**Authors:** Anastasia S. Lambrou, Erin South, Eliza S. Ballou, Clinton R. Paden, James A. Fuller, Stephen M. Bart, Deena M. Butryn, Ryan T. Novak, Sean D. Browning, Amy E. Kirby, Rory M. Welsh, Daniel M. Cornforth, Duncan R. MacCannell, Cindy R. Friedman, Natalie J. Thornburg, Aron J. Hall, Laura J. Hughes, Barbara E. Mahon, Demetre C. Daskalakis, Nirav D. Shah, Brendan R. Jackson, Hannah L. Kirking

**Affiliations:** ^1^Coronavirus and Other Respiratory Viruses Division, National Center for Immunization and Respiratory Diseases, CDC; ^2^General Dynamics Information Technology, Inc., Atlanta, Georgia; ^3^Office of the Director, National Center for Immunization and Respiratory Diseases, CDC; ^4^Goldbelt LLC, Anchorage, Alaska; ^5^Division of Global Health Protection, Global Health Center, CDC; ^6^Division of Global Migration Health, National Center for Emerging and Zoonotic Infectious Diseases, CDC; ^7^Office of the Director, Global Health Center, CDC; ^8^Division of Infectious Disease Readiness and Innovation, National Center for Emerging and Zoonotic Diseases, CDC; ^9^Office of the Director, CDC.

**Summary**

**What is already known about this topic?**

Early detection of emerging SARS-CoV-2 variants is critical to assessing risk, providing clear and timely communication messages, and coordinating public health action. CDC tracks SARS-CoV-2 variants using multiple approaches, including genomic, wastewater, traveler-based, and digital public health surveillance.

**What is added by this report?**

The SARS-CoV-2 variant BA.2.86 was first reported in August 2023. CDC used a multicomponent surveillance approach to track its global spread. It has been reported in 32 countries as of October 23, 2023.

**What are the implications for public health practice?**

An early warning multicomponent surveillance approach can provide valuable actionable information not only for novel SARS-CoV-2 variants but also for future pathogen threats.

## Abstract

Early detection of emerging SARS-CoV-2 variants is critical to guiding rapid risk assessments, providing clear and timely communication messages, and coordinating public health action. CDC identifies and monitors novel SARS-CoV-2 variants through diverse surveillance approaches, including genomic, wastewater, traveler-based, and digital public health surveillance (e.g., global data repositories, news, and social media). The SARS-CoV-2 variant BA.2.86 was first sequenced in Israel and reported on August 13, 2023. The first U.S. COVID-19 case caused by this variant was reported on August 17, 2023, after a patient received testing for SARS-CoV-2 at a health care facility on August 3. In the following month, eight additional U.S. states detected BA.2.86 across various surveillance systems, including specimens from health care settings, wastewater surveillance, and traveler-based genomic surveillance. As of October 23, 2023, sequences have been reported from at least 32 countries. Continued variant tracking and further evidence are needed to evaluate the full public health impact of BA.2.86. Timely genomic sequence submissions to global public databases aided early detection of BA.2.86 despite the decline in the number of specimens being sequenced during the past year. This report describes how multicomponent surveillance and genomic sequencing were used in real time to track the emergence and transmission of the BA.2.86 variant. This surveillance approach provides valuable information regarding implementing and sustaining comprehensive surveillance not only for novel SARS-CoV-2 variants but also for future pathogen threats.

## Introduction

CDC uses a diverse, multicomponent surveillance approach to track the emergence of new and potentially significant SARS-CoV-2 variants across the United States and globally. These surveillance systems include genomic, wastewater, traveler-based, and digital public health surveillance, which complement other traditional public health surveillance systems (Box). The implementation of a multicomponent approach optimizes timely collection of the best available data, because each individual surveillance method might not capture all COVID-19 cases, and not all specimens will undergo genomic sequencing.

BOX Components of SARS-CoV-2 surveillance* — United States, 2023

**Vital records**

- NVSS

**Health care facilities**

- NREVSS
- NHSN
- NSSP
- Unified Hospital Data Set
- NVSN
- COVID-NET
- National SARS-CoV-2 genomic surveillance

**Community**

- ICATT
- National SARS-CoV-2 genomic surveillance

**Traveler**

- TGS

**Wastewater**

- NWSS
- Academic, private, and local jurisdictional wastewater surveillance activities

**Digital**

- News media
- Social media
- Global public health partner reports
- Public genomic data repositories
- GISAID

**Abbreviations:** COVID-NET = COVID-19–Associated Hospitalization Surveillance Network; GISAID = Global Initiative on Sharing All Influenza Data; ICATT = Increasing Community Access to Testing; NHSN = National Healthcare Safety Network; NREVSS = National Respiratory and Enteric Virus Surveillance System; NSSP = National Syndromic Surveillance Program; NVSN = New Vaccine Surveillance Network; NVSS = National Vital Statistics System; NWSS = National Wastewater Surveillance System; TGS = Traveler-based Genomic Surveillance Program.

* More detailed clinical and epidemiologic data are available from vital records and health care facilities and other more traditional surveillance systems; however, these data are less timely. Data from community, travel, wastewater, and digital surveillance are less illness- and infection-specific but are timelier and can provide early warning.

Each surveillance component provides distinct information, that, when considered together, enable robust situational awareness for early warning signals and support epidemiologic characterization if more widespread transmission is established. The SARS-CoV-2 variant BA.2.86, first detected in August 2023, has more than 30 mutations in the spike protein compared with other currently circulating variants. This sequence divergence of BA.2.86 suggested potentially reduced antibody protection from previous SARS-CoV-2 infection and vaccination, especially before early laboratory-based evaluations were conducted. Consequently, CDC is actively monitoring BA.2.86 to guide public health actions and surveillance efforts (*1*). Continued variant tracking and further evidence, such as real-word evaluations, are needed to understand the full public health impact of BA.2.86. This report highlights the use of a diverse, multicomponent surveillance system for early warning, and describes how this approach has informed the response to the SARS-CoV-2 BA.2.86 variant.

## Methods

### Surveillance System Data

Data from four early warning surveillance systems were analyzed in this report: 1) National SARS-CoV-2 genomic surveillance,^†^ 2) Traveler-based Genomic Surveillance (TGS), 3) the National Wastewater Surveillance System (NWSS), and 4) digital public health surveillance. National SARS-CoV-2 genomic surveillance comprises three different sequence sources that are combined and modeled to create weighted estimates of variant proportions for every 2-week period. These data are also used to create Nowcast estimates, which are model-based projections of variant proportions for the most recent 2-week period (*2*). CDC’s TGS program collects nasal swab samples from volunteer international travelers arriving at six major U.S. international airports (*3*) from more than 135 countries.^§^ CDC’s NWSS operates across 50 states and two U.S. territories covering sewer sheds that service 40% of the U.S. population^¶^ (*4*). SARS-CoV-2 sequences from global genomic surveillance systems are uploaded to the National Center for Biotechnology’s Information Sequence Read Archive (NCBI SRA)** and Global Initiative on Sharing All Influenza Data (GISAID).^††^ Digital public health surveillance includes monitoring of global public genomic data repositories such as NCBI and GISAID and also includes monitoring other digital content such as news media, social media, and global event-based and public health partner reports.^§§^

### Analyses

For this analysis, BA.2.86 reports from digital public health surveillance were collected and confirmed by a CDC team. Sequences in public databases and corresponding metadata were examined daily from NCBI and GISAID. These data were analyzed using descriptive statistics and used for geographic and temporal mapping. A more detailed data analysis was conducted using sequences reported during the first 2 weeks after the emergence of the BA.2.86 variant to describe the earliest available data in more detail. In addition, differences in lag time between specimen collection and reporting dates were calculated and compared using global repository metadata. Analyses were conducted in R (version 4.1.3; R Foundation). Early public health actions that were taken as a result of these early warning surveillance data are also described in this report to illustrate how these data were used in real time. This activity was reviewed by CDC, deemed not research, and conducted consistent with applicable federal law and CDC policy.^¶¶^

## Results

### Tracking BA.2.86 Emergence

The first global BA.2.86 case was submitted to GISAID from Israel on Sunday, August 13, 2023 (Table). The next day the second and third BA.2.86 cases were reported by Denmark; these two cases were not epidemiologically linked to one another. On August 17, the United States reported the fourth BA.2.86 case (in Michigan), and on the same day, NWSS reported the first U.S. BA.2.86 detection in an Ohio wastewater sample, which had been collected on July 30. During the following 10 days, nine additional BA.2.86 cases were reported. Additional countries reporting respiratory cases included the United Kingdom and South Africa, and Switzerland and Denmark reported wastewater detections. On August 10, BA.2.86 was detected in a sample collected from a TGS participant traveling from Japan arriving at Dulles International Airport (near the District of Columbia) (*5*) and confirmed on August 20 (Figure). On August 26, Ohio reported a case from a patient sample collected on July 29. The patient had been in the same area where the first wastewater detection was reported 9 days earlier. Although systematic case investigations were not conducted, at least one of the early U.S. cases was confirmed to have no history of international travel.

**TABLE:** Reported global detections of the SARS-CoV-2 BA.2.86 variant in the 2 weeks after initial report* (N = 14) — worldwide, August 13–26, 2023

Specimen source/Country	Collection date	Report date	Interval, days^†^	Surveillance component
**Respiratory specimens**				
Israel	Jul 31	Aug 13	13	Digital public health
Denmark	Jul 24	Aug 14	21	Digital public health
Denmark	Jul 31	Aug 14	14	Digital public health
United States (Michigan)	Aug 3	Aug 17	14	Respiratory specimen genomic
United Kingdom (England)	Aug 13	Aug 18	5	Digital public health
Denmark	Aug 7	Aug 19	12	Digital public health
United States/Japan	Aug 10	Aug 21	11	Traveler-based genomic
South Africa	Jul 24	Aug 22	29	Digital public health
South Africa	Jul 28	Aug 22	25	Digital public health
Denmark	Aug 14	Aug 25	11	Digital public health
United States (Ohio)	Jul 29	Aug 26	28	Respiratory specimen genomic
**Wastewater**				
United States (Ohio)	Jul 30	Aug 17	18	Wastewater genomic
Switzerland	Aug 4	Aug 23	19	Wastewater genomic
Denmark	Missing	Aug 25	—	Wastewater genomic

* August 13, 2023.

**^†^** Number of days from respiratory specimen or wastewater sample collection to public report.

**FIGURE:**
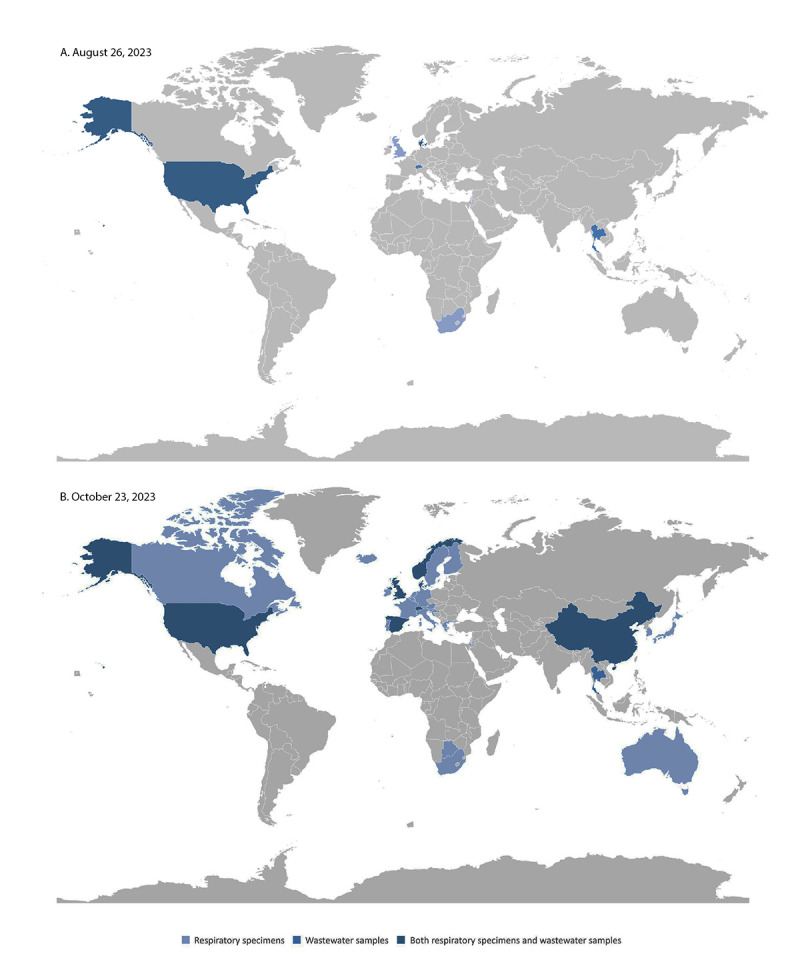
SARS-CoV-2 BA.2.86 variant detection in respiratory specimens and wastewater samples, by country — worldwide, August 26 (A) and October 23, 2023 (B)

The earliest respiratory specimen collection date for a BA.2.86 detection was collected in Denmark on July 24 (the second global reported BA.2.86 detection); the earliest U.S. specimen collection date was July 29 (the second U.S. reported BA.2.86 detection). Among the 11 BA.2.86 sequence detections reported in the 2 weeks after the first reported detection, the median lag time between specimen collection and sequence reporting for respiratory detections was 14 days (range = 5–29 days), including the TGS sample, for which the lag time from collection to reporting was 10 days. The median lag time from two of the three earliest wastewater detections to sequence reporting was 18 days (the collection date was missing for the third specimen).*** 

After these initial 14 detections, BA.2.86 was detected through respiratory or wastewater samples in at least 32 countries worldwide, across five continents. As of October 23, using publicly available data from 945 specimens in GISAID, the median lag time from specimen collection date to report date is 15 days (range = 4–53 days) (Supplementary Figure 1, https://stacks.cdc.gov/view/cdc/134230). As of October 23, 2023, BA.2.86 accounted for <1% of circulating variants in the United States (Supplementary Figure 2, https://stacks.cdc.gov/view/cdc/134231).^†††^

### Public Health Response

Data related to the early detection of BA.2.86 enabled rapid risk communication, viral isolation and characterization, and cross-coordination for public health action. Rapid risk communication was released through timely, web-based Respiratory Virus Updates^§§§^ (weekly updates on the respiratory illness season) to alert public health partners and the public. Early detection of BA.2.86 through genomic surveillance also facilitated collaborations between sequencing laboratories and CDC. Residual virus samples were shared with CDC laboratories for isolation in viral culture, early characterization, and laboratory-based neutralization studies to better understand the potential impact of immune escape. High-quality, rapidly generated BA.2.86 sequences facilitated the understanding of the wide geographic distribution of the lineage and aided early laboratory-based and computer-modeled studies predicting immune escape.

## Discussion

Despite decreased SARS-CoV-2 sequencing resulting from changing COVID-19 testing practices, U.S. genomic surveillance systems detected BA.2.86, a novel SARS-CoV-2 lineage circulating at very low levels. Using multiple surveillance systems enhanced early detection, tracking, and characterization of emerging SARS-CoV-2 variants. The first U.S. detection of BA.2.86 was identified through a health care facility specimen that was sent to CDC by the state laboratory for isolation and further characterization. Successful virus isolation at CDC allowed for the sharing of BA.2.86 isolates with other laboratories. TGS detected BA.2.86 in a sample from a traveler returning to the United States who was likely infected while abroad (*5*). NWSS facilitated BA.2.86 early warning in additional areas, and wastewater surveillance was a leading indicator in Ohio where BA.2.86 was identified 9 days before a respiratory sequence was reported in the same area.

Specimen collection dates support that BA.2.86 was likely beginning to circulate in the United States before the end of July 2023. Currently, BA.2.86 has not become predominant but is likely circulating across the United States at low levels. Preliminary laboratory research findings indicate that existing antibodies from previous SARS-CoV-2 infection or vaccination are effective in neutralizing BA.2.86 but real-world human outcome data are also needed to better understand the impacts of preexisting immunity^¶¶¶^^,^**** (*6*).

Early warning of SARS-CoV-2 variant detection enables timely assessments of risk, mobilization of resources, clear and timely communication, and coordinated public health action (*7*). The complementary surveillance systems provided critical data and specimens for culture, treatment effectiveness evaluation, and will facilitate the development of other treatments, as needed. Integrating pathogen genomic sequencing throughout the different surveillance system components added important molecular resolution for tracking variant emergence and transmission dynamics. Digital public health surveillance can provide a signal to enhance and focus other surveillance systems toward detection of new variants. Global information-sharing and partnerships for early warning also played an important role; these systems and partnerships are crucial in light of the decrease in specimen sequencing.

Other existing surveillance systems^††††^ might become critical to monitoring the impacts of BA.2.86. If circulation increases, epidemiologic data related to relative transmissibility, disease severity, and vaccine and therapeutic effectiveness will be important to understanding this variant’s impact on human health. If BA.2.86 exceeds 1% of circulating variants within the United States, it will be reported through CDC’s Nowcast estimates over time and by region. If BA.2.86 circulation expands to represent a significant proportion of circulating variants in the United States, other complementary COVID-19 surveillance systems that capture detailed laboratory- and patient-level data can be tracked in parallel to understand epidemiologic impacts. If new data become available that result in heightened concern, CDC can launch epidemiologic field studies on transmissibility and severity.

### Limitations

The findings of this report are subject to at least five limitations. First, data analyzed from complementary surveillance systems included varying levels of geographic, epidemiologic, clinical, and demographic information. Links between surveillance data and epidemiologic and clinical data necessary to guide action are often missing, limiting the level of analysis performed. In addition, unequal levels of global sequencing capacity and funding also limit understanding of geographic spread. Second, digital public health surveillance methods employed both informal and formal manual data gathering, which was resource-intensive. Third, global genomic surveillance is limited by the variable lag times between specimen collection and reporting, which can impact real-time actionability. Fourth, standardized national methods for genomic sequence (or partial sequence) data reporting into different public repositories are lacking; this limitation is especially apparent for wastewater sequences. Finally, data quality, reporting, and aggregation standards are needed for multicomponent pathogen genomic surveillance.

### Implications for Public Health and Preparedness

The emergence of BA.2.86 has highlighted the importance of early detection through multiple, complementary surveillance systems involving diverse approaches, populations, and specimen types. These systems can be further improved by addressing timeliness, improving understanding of both the strengths and limitations of each system, and increasing cross-public health coordination and action. Leveraging, maintaining, and prioritizing these robust, multipurpose public health surveillance systems require sustained financial resources.

Early detection data are more actionable as the lag time between specimen collection and reporting of results decreases, and when more clinical and epidemiologic data are available. Innovations in pathogen testing and genomic sequencing, capacity building, and reporting systems can support earlier public health action. New technology is also needed from the private sector to offer less expensive, targeted, and more sustainable products (e.g., cheaper, faster diagnostic tests) to support the future of public health surveillance. Continuous, automated data scraping^§§§§^ for early warning signs (e.g., robust event-based surveillance) can more efficiently alert health authorities of global events to guide preparation measures. Complementary surveillance systems in place for early warning can be used for other known and novel public health threats. As public health and surveillance advancements continue, the deployment of multiple innovations to strengthen early warning, preparedness, and response will be critical.

## Footnotes

* These authors contributed equally to this report.

† National SARS-CoV-2 genomic surveillance comprises three genomic sequencing sources of data from respiratory virus specimen collection: 1) national SARS-CoV-2 strain surveillance, 2) commercial contract laboratory sequencing, and 3) sequences from public health, academic, and clinical laboratories that are tagged as baseline surveillance in public genomic data repositories. https://www.cdc.gov/coronavirus/2019-ncov/variants/cdc-role-surveillance.html

§ CDC TGS program operates in Los Angeles (LAX), Newark (EWR), New York (JFK), San Francisco (SFO), Seattle (SEA), and Washington D.C./Dulles (IAD) airports. https://wwwnc.cdc.gov/travel/page/travel-genomic-surveillance

¶ CDC National Wastewater Surveillance System operates in all 50 U.S. states, District of Columbia, and two territories (Puerto Rico and Guam). https://www.cdc.gov/nwss/wastewater-surveillance.html

**
https://www.ncbi.nlm.nih.gov/sra

††
https://gisaid.org/

§§ Digital public health surveillance manual formal data collection included structured data pulls from public genomic repositories. Manual informal data collection methods included monitoring social media, such as X, for updated genomic surveillance news.

¶¶ 45 C.F.R. part 46.102(l)(2), 21 C.F.R. part 56; 42 U.S.C. Sect.241(d); 5 U.S.C.0 Sect.552a; 44 U.S.C. Sect. 3501 et seq.

***
https://twitter.com/SSI_dk/status/1695027179711533235

†††
https://covid.cdc.gov/covid-data-tracker/#variant-proportions

§§§
https://www.cdc.gov/respiratory-viruses/whats-new/index.html

¶¶¶
https://www.biorxiv.org/content/10.1101/2023.09.04.556272v1

****
https://www.biorxiv.org/content/10.1101/2023.09.07.556636v1#:~:text=The%20pseudovirus%20assay%20showed%20that,due%20to%20the%20increased%20infectivity

†††† These surveillance systems include National Syndromic Surveillance Program (https://www.cdc.gov/nssp/overview.html) capturing emergency department visits, National Respiratory and Enteric Virus Surveillance System (https://www.cdc.gov/surveillance/nrevss/index.html) capturing proportion of COVID nucleic acid amplification test/polymerase chain reaction test positivity, National Hospital Sentinel Network (https://www.cdc.gov/nhsn/index.html) capturing hospitalization data, Unified Hospital Data Set, New Vaccine Surveillance Network (https://www.cdc.gov/surveillance/nvsn/index.html), and COVID-19–Associated Hospitalization Surveillance Network (https://www.cdc.gov/coronavirus/2019-ncov/covid-data/covid-net/purpose-methods.html).

§§§§ Data scraping is the process of extracting data from the Internet.
